# Stromal cell-derived factor loaded co-electrospun hydrophilic/hydrophobic bicomponent membranes for wound protection and healing†

**DOI:** 10.1039/d0ra04997b

**Published:** 2020-12-24

**Authors:** Robin Augustine, Syed Raza ur Rehman, Joshy K. S., Anwarul Hasan

**Affiliations:** Department of Mechanical and Industrial Engineering, College of Engineering, Qatar University – 2713 Doha Qatar ahasan@qu.edu.qa; Biomedical Research Center, Qatar University – 2713 Doha Qatar

## Abstract

Chronic wounds are one of the key concerns for people with diabetes, frequently leading to infections and non-healing ulcers, and finally resulting in the amputation of limbs/organs. Stromal cell-derived factor 1 (SDF1) is a major chemokine that plays a significant role in tissue repair, vascularization, and wound healing. However, the long-term sustained delivery of SDF1 in a chronic wound environment is a great challenge. In order to facilitate the sustained release of SDF1 in diabetic wounds, it could be incorporated into wound-healing patches. Herein, we report the fabrication of a hydrophilic/hydrophobic bicomponent fiber-based membrane, where SDF1 was encapsulated inside hydrophilic fibers, and its applicability in wound healing. A co-electrospinning technique was employed for the fabrication of polymeric membranes where PVA and PCL form the hydrophilic and hydrophobic components, respectively. Morphological analysis of the developed membranes was conducted *via* scanning electron microscopy (SEM). The mechanical strength of the membranes was investigated *via* uniaxial tensile testing. The water uptake capacity of the membranes was also determined to understand the hydrophilicity and exudate uptake capacity of the membranes. To understand the proliferation, viability, and migration of skin-specific cells in the presence of SDF1-loaded membranes, *in vitro* cell culture experiments were carried out using fibroblasts, keratinocytes, and endothelial cells. The results showed the excellent porous morphology of the developed membranes with distinguishable differences in fiber diameters for the PVA and PCL fibers. The developed membranes possessed enough mechanical strength for use as wound-healing membranes. The co-electrospun membranes showed good exudate uptake capacity. The controlled and extended delivery of SDF1 from the developed membranes was observed over a prolonged period. The SDF1-loaded membranes showed enhanced cell proliferation, cell viability, and cell migration. These biocompatible and biodegradable SDF1-loaded bicomponent membranes with excellent exudate uptake capacity, and cell proliferation and cell migration properties can be exploited as a novel wound-dressing membrane aimed at chronic diabetic wounds.

## Abstract 2

Co-electrospun membranes containing SDF1 with micro/nano topography and hydrophilic/hydrophobic surface properties promote the migration and proliferation of mammalian cells that are relevant to wound healing.

## Graphical abstract

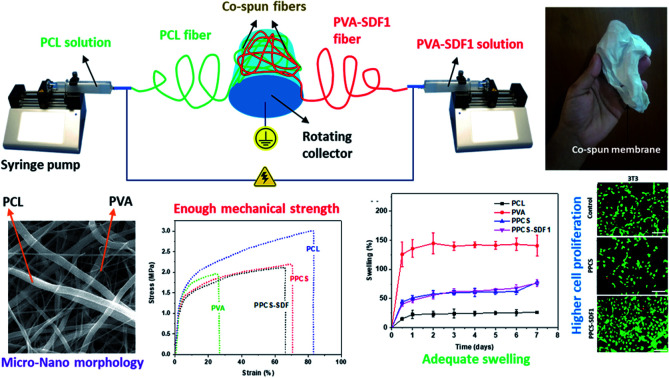

## 1. Introduction

Diabetes mellitus is a common metabolic disorder affecting 1–2% of the total global population with a noticeable health risk and economic burden.^1^ About 15% of diabetic patients show persistent wounds in their lower extremities, which frequently necessitate organ or limb amputation.^2,3^ Wounds in diabetic patients have comparatively extended healing periods, due to persistent inflammation, poor vascularisation, unusual production of exudate and high bacterial load in the wound.^4^ Multiple hurdles at a cellular level, such as lack of cell viability, cell migration and cell proliferation, are observed in chronic diabetic wounds.^5^ These often result in the destruction of vascularization, hindrance in wound contraction and the development of non-healing diabetic ulcers.

Various studies have clearly indicated the role of growth factors, like epidermal growth factor (EGF),^6^ connective tissue growth factor (CTGF),^7^ vascular endothelial growth factor (VEGF),^8^ platelet derived growth factor (PDGF)^9^ and stromal cell-derived factor 1 (SDF1),^10^ during various stages of wound contraction, re-epithelialization and wound-remodelling. Such growth factors are being effectively utilized as appropriate active agents for reducing the chronicity and promoting the healing of diabetic wounds.^11^ SDF1 was originally found in human bone marrow, and has recently been identified to be generated in stromal tissues in several tissues.^12^ A study conducted in diabetic rats indicated that the inhibition of SDF1 activity can further delay the wound healing process in diabetic patients.^13^ For instance, loading of SDF1 in wound dressing materials accelerated the healing of diabetic wounds through the enhancement of cell proliferation, and promotion of cell migration^14^ and angiogenesis.^15^ Earlier reports also suggest that the differentiation, migration and proliferation of endothelial cells are promoted by SDF1, and these are considered as important steps of vascularization in the healing wound.^16^ Nevertheless, the bench-to-bed side translation of SDF1-based medications is greatly hampered by its inactivity in the extremely oxidative environment of diabetic wounds. This limitation demands a platform to protect it in the wound environment and facilitate its controlled delivery in the wound bed.^17,18^ Hence, the incorporation of SDF1 in polymeric wound healing membranes is highly promising, as it facilitates controlled release, achieves the desired cell proliferation, cell migration and neovascularization, and finally leads to fast healing of diabetic wounds.^19^

Natural or synthetic polymer-based hydrogels can find applications as advanced wound dressing materials due to their high exudate uptake capacity, biodegradability, biocompatibility and processability. Poly(vinyl alcohol) (PVA) is one among them that can be used as a carrier matrix for the effective encapsulation and delivery of active agents including growth factors.^20^ Owing to the advantages of PVA, like biodegradability, ease of processability and low cost, it has received much attention for numerous healthcare applications^21^ and especially as a wound dressing material.^22,23^ Due to their very high hydrophilic properties, PVA-based biomaterials are not mechanically very stable. In order to overcome this limitation of PVA, we aim to co-electrospin it with another mechanically stable hydrophobic polymer. For this, we selected polycaprolactone (PCL), which is already recognized as a promising candidate for various healthcare applications such as wound healing membranes/dressings, drug delivery systems and tissue engineering scaffolds.^24–26^ PCL has good biocompatibility,^27,28^ biodegradability^29^ and relatively high hydrophobicity.^30^ Patches based on PCL and PVA combinations are used for wound healing applications with promising outcomes.^31^

Electrospinning is a widely accepted method for the generation of nonwoven highly porous submicron fiber-based membranes for diverse applications in the biomedical field.^32–36^ Co-electrospinning (or co-spinning) is a highly promising approach employed to produce membranes composed of fibers from multiple polymers, even with completely different physicochemical properties.^37^ There are some recent reports regarding the fabrication and evaluation of co-electrospun PCL and PLA membranes for wound healing applications.^38,39^ Here, co-spinning was applied for the fabrication of wound dressing membranes composed of PCL fibers and PVA fibers encapsulated with SDF1.

In this study, we described the fabrication of co-spun fiber-based membranes loaded with the growth factor, SDF1, for possible applications in diabetic wound healing. The primary focus of the present investigation was to design membranes composed of SDF1 loaded-PVA fibers that facilitate the sustained release of SDF1 over an extended period of time and PCL fibers that provide mechanical stability to the membrane.

## 2. Materials and methods

### 2.1. Materials

PVA (Avg. *M*_w_: 8 × 10^4^–9 × 10^4^), PCL (Avg. *M*_w_: 8 × 10^3^ × 10^4^), SDF1, and 3-(4,5-dimethylthiazol-2-yl)-2,5-diphenyltetrazolium bromide (MTT) were purchased from Sigma-Aldrich. Dulbecco's Modified Eagle's Medium (DMEM), phosphate buffered saline (PBS) and penicillin-streptomycin solution were purchased from Gibco. Phalloidin, 4′,6-diamidino-2-phenylindole (DAPI) and the live/dead assay kit were purchased from Invitrogen.

### 2.2. Fabrication of SDF1-loaded co-spun membranes

**Fig. 1:**
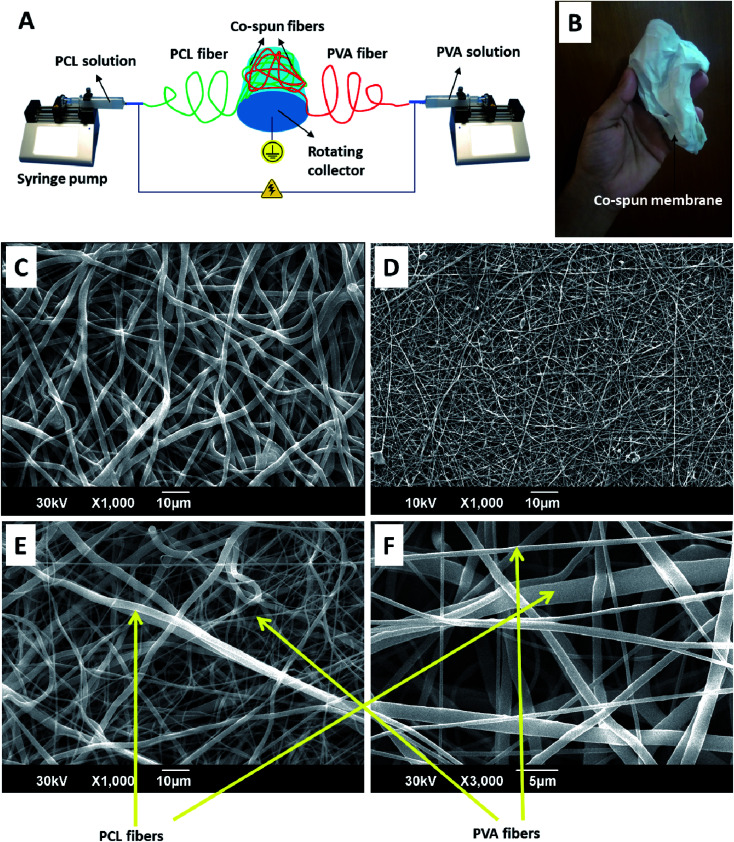
A schematic diagram representing the fabrication process of the PVA-PCL co-spun fiber-based (PPCS) membranes (A). A digital photograph showing the visual appearance of the membrane (B). SEM images showing the morphologies of the PCL (C), PVA (D), and PPCS (E) membranes. A higher magnification image of PPCS showing the component PCL and PVA fibers (F).

Wound healing membranes were fabricated by mixing the desired quantity of SDF1 in PVA matrix and ultra-sonicating for about 15 minutes to obtain a homogeneous solution in PBS. The required concentration of SDF1 was chosen based on preliminary *in vitro* studies that were performed using keratinocytes and fibroblasts (see the ESI Fig. S1 and S2†). To make the PVA solution, PVA powder was dissolved in heated ultrapure water to get a 10% w/w solution. The PVA solution was cooled to room temperature before the addition of SDF1 to prevent the loss of its bioactivity. The SDF1 concentration in PVA was maintained as 0.01% w/w (with respect to PVA weight, a total amount of 50 μg of SDF1 was loaded per sample produced from 0.5 g of PVA). The PCL solution (12%) was obtained by dissolving PCL powder in a DCM/DMF (1 : 9 ratio) mixture. The spinning set-up consisted of two syringe pumps (infusion), a high voltage DC power supply (15 kV) and a rotating drum collector (1000 rpm). The drum collector was placed between the syringe pumps in such a way that the fibers will be deposited on either side of the collector (Fig. 1). The two separate syringes (10 mL) containing PCL and PVA solutions (5 mL each with and without SDF1) were attached to the syringe pumps. The flow rate of both the PCL and PVA solutions was 1 mL h^−1^. Electrospinning was performed at a 10 cm fixed tip-to-collector distance. Crosslinking of the membranes, except for the single component PCL membranes, was performed using 2 M glutaraldehyde solution by a reported method.^40^ Electrospun PCL or PVA membranes alone were also generated at similar spinning parameters. The electrospun PCL membranes, electrospun PVA membranes, co-spun membranes and co-spun PVA-PCL membranes loaded with SDF1 were denoted as PCL, PVA, PPCS and PPCS-SDF1, respectively.

### 2.3. Physico-mechanical characterization of the membranes

#### 2.3.1. SEM analysis

A scanning electron microscope (FEI) was utilized to analyze the ultrafine morphology of the developed membranes. The samples were coated with gold and analyzed at 10 kV accelerating voltage. The fiber diameters were calculated using ImageJ software.

#### 2.3.2. Mechanical properties of the membranes

The tensile properties of the PCL, PVA, PPCS and PPCS-SDF1 membranes were measured by a uniaxial universal testing machine (Tinus Olsen H50 KT) based on the standard protocol (ASTM D 882). 6 × 1 cm^2^ rectangular samples were used for the measurements. The gauge length was fixed to 3 cm, and an operating rate of 1 mm min^−1^ and mechanical loading of 500 N were applied on the membrane samples.

#### 2.3.3. Water contact angle measurements

The water contact angle on the surface of the membranes was measured by a contact angle measurement system equipped with a digital camera. Each sample was kept flat on the platform of the contact angle system, a water droplet was placed over the sample, a series of images of the water droplets were captured by the camera and the contact angle was measured from the images using ImageJ software.

#### 2.3.4. Exudate uptake capacity of the membranes

The ability of the PCL, PVA, PPCS and PPCS-SDF1 samples to absorb exudate when applied on wounds was examined by swelling studies. The samples were cut into 2 × 2 cm^2^ pieces. The weighed samples were placed in PBS at room temperature for up to 7 days. The samples were blotted using Whatman No. 1 filter paper to eliminate the adsorbed water on the sample surface. The water uptake capacity of the membranes was determined by using eqn (1) based on the dry and wet weights.

1 Swelling (%) = [(wet weight − dry weight)/dry weight] × 100

#### 2.3.5. Release of SDF1 from the membranes

The release study was carried out according to the reported procedure with slight modifications.^41^ Briefly, 5 mg samples of the weighed membranes were immersed in 12-well cell culture plates containing serum free DMEM medium (1 mL) and placed in a CO_2_ incubator with a 5% CO_2_ supply at 37 °C. At regular intervals, the release medium (400 μL) was taken out from each well and replaced with fresh medium. The protein content in the release medium was estimated using a protein estimation kit according to the manufacturer's protocol (Pierce BCA, Thermo Scientific).

### 2.4. *In vitro* cell culture studies

#### 2.4.1. Membrane preparation and cell seeding

Prior to the seeding of mammalian cells on PCCS and PCCS-SDF1 membranes, they were cut into 1 × 1 cm sized pieces and sterilized by alcohol (70% for 10 min) treatment and UV (10 min) exposure. Mouse 3T3 fibroblast cells (ATCC CRL-1658, Passage-28), human HaCaT keratinocytes (Passage-34) and human EA.hy926 endothelial cells (ATCC CRL-2922, Passage-17) were used for this study. About 2 × 10^4^ cells per sample were seeded on each sample and cultured in 24 well cell culture plates. Wells seeded with cells but without any samples were maintained as controls. All the cells were cultured in DMEM with 10% fetal bovine serum and 1% penicillin/streptomycin solution. The cell seeded membranes were incubated for up to 5 days at 37 °C with 5% CO_2_ supply.

#### 2.4.2. Determination of cell viability after treatment with the membranes

##### 2.4.2.1 Live/dead test

To visualize live and dead cells in the samples, a live/dead assay was performed according to the manufacturer's protocol (Invitrogen, USA) at the 5^th^ day of cell culture. The cells were imaged using a fluorescent microscope (Olympus, FV300).

##### 2.4.2.2 MTT assay

An MTT assay was conducted to determine the cell viability after culturing with neat and SDF1-loaded membranes. About 2 × 10^4^ cells (either 3T3 fibroblasts, HaCaT keratinocytes or EA.hy926 endothelial cells) were cultured on 24-well plates and after 24 h incubation, 1 × 1 cm sized sterilized samples were placed over the cells. The plates were incubated for up to 5 days in an incubator with 5% CO_2_ supply, and MTT assays were carried out based on the manufacturer's procedure (Invitrogen, USA). The medium was replaced with fresh medium every 48 h to avoid nutrient depletion and subsequent cell death. The percentage of cell viability was calculated in accordance with eqn (2).

2 Cell viability (%) = (OD of sample/OD of control) × 100

#### 2.4.3. *In vitro* scratch wound healing test

An *in vitro* scratch test was conducted on the monolayer 3T3 fibroblasts, HaCaT keratinocytes or EA.hy926 endothelial cells as described in earlier work.^40^ Briefly, the cells were cultured in 12 well plates at an initial seeding density of 5 × 10^4^ cells per well. After getting confluent growth of the cells on the culture plate, a scratch was introduced with the help of a 100 μL pipette tip. The cells were then incubated with pre-sterilized scaffolds (1 × 1 cm). Images of the scratched area before sample placement and after 24 h of incubation were taken with a microscope (Olympus, FV300). The gaps between the scratch edges were determined using ImageJ software and the scratch healing (%) was determined in accordance with eqn (3).

3 Scratch healing (%) = (*Wd*^0^ − *Wd*^*t*^)/*Wd*^0^ × 100

where *Wd*^0^ and *Wd*^*t*^ respectively are the space between the scratch boundaries before and after the incubation time ‘*t*’, respectively.

### 2.5. Statistical analysis

All of the tests were performed in triplicate, the mean values of the obtained results of each group were calculated and the statistical significance was determined from Analysis of Variance (ANOVA). *P* < 0.05 (denoted with * in the figures) was considered as statistically significantly different from the other group of comparison.

## 3. Results

### 3.1. Morphology of the developed membranes

**Fig. 2:**
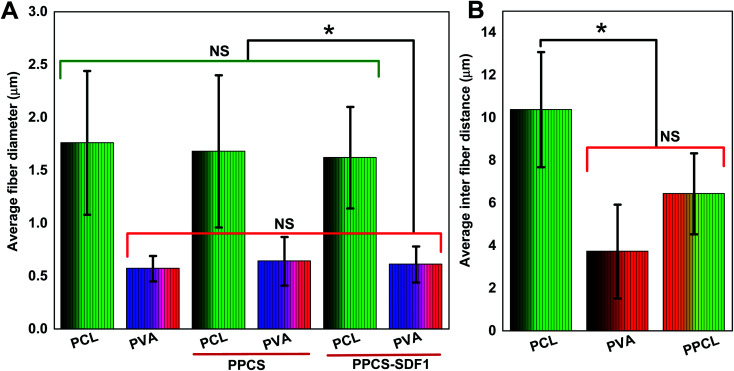
Average fiber diameters and inter-fiber distances of the PCL, PVA, PPCS, and PPCS-SDF1 membranes. Average fiber diameters of the PCL, PVA, PPCS, and PPCS-SDF1 membranes (A). Average inter-fiber distances of the PCL, PVA, and PPCL membranes (B). (*) indicates *p*-values where a statistically significant difference (*p* ≤ 0.05) is observed. NS indicates no statistically significant difference.

The obtained membranes were highly flexible with a thin cloth-like visual appearance (Fig. 1B). The microscale morphology of the samples was analyzed using the SEM technique. The single component PCL and PVA membranes were composed of uniform individual fibers without any bead formation or irregularities (Fig. 1C and D). From the SEM images, it was clear that the co-spun membranes were composed of PCL and PVA fibers, which were clearly distinguishable based on the difference in fiber diameter (Fig. 1E, F, Fig. 2). Specifically, the average fiber diameters of the single component PCL and PVA membranes were calculated as 1.76 ± 0.68 μm and 0.57 ± 0.12 μm, respectively. The average fiber diameters of the component PCL and PVA fibers in the co-spun membranes were 1.68 ± 0.72 μm and 0.64 ± 0.23 μm, respectively. The surface of the obtained fibers was relatively smooth and the fibers on the co-spun membranes did not show any beads or irregularities. There was no substantial variation between the fiber diameters or the fiber morphology of the PPCS and PPCS-SDF1 membranes. In order to quantify the amount of PVA and PCL components in the co-electrospun membranes, we selectively removed one of the components (here, PCL was removed) by dissolving it in a suitable solvent (acetone). The obtained results show that (Table S1†) the average PVA and PCL contents in the co-electrospun fibrous membranes were 44.06 ± 3.16 and 56.27 ± 2.76, respectively.

### 3.2. Tensile properties of the membranes

**Fig. 3:**
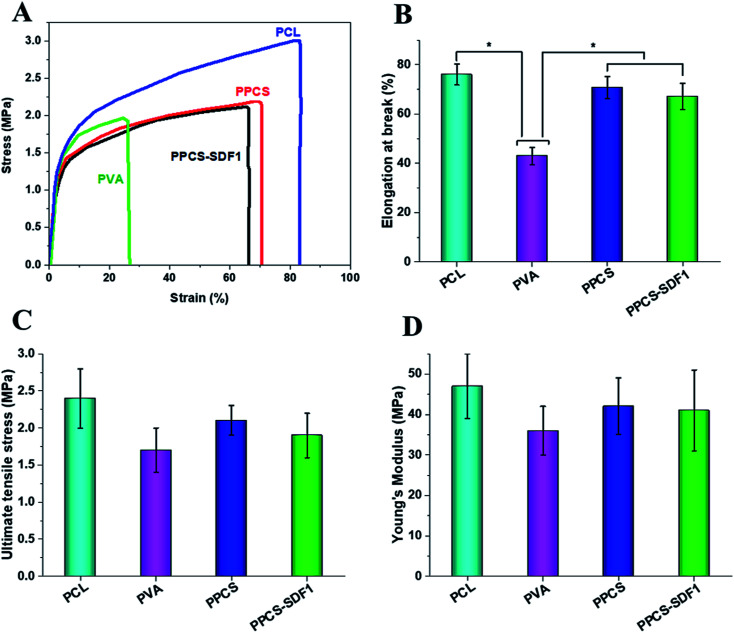
Uniaxial tensile testing results of the PCL, PVA, PPCS, and PPCS-SDF1 membranes. Representative stress–strain curves (A), mean value of elongation at break (B), mean value of ultimate tensile stress (C), and mean value of Young's modulus (D) of PCL, PVA, PPCS, and PPCS-SDF1 membranes. (*) indicates *p*-values (*p* ≤ 0.05) where a statistically significant difference was observed between compared groups.

The tensile strength and elasticity of the developed neat and SDF1-loaded membranes were measured by uniaxial tensile testing and the results are shown in Fig. 3A–D. Representative stress–strain graphs are given in Fig. 3A, which show a linearly elastic region at low strain and a plastic deformation before elongation at break for all the tested samples. The pure PCL membranes exhibited a fracture strain of 78 ± 5.1% and ultimate tensile stress of 2.4 MPa. The PCL membranes showed an average Young's modulus of 46.21 ± 11.43 MPa. In contrast, the PVA membranes displayed an elongation at break of 42.5 ± 4.6% and ultimate tensile stress of 1.7 ± 0.7 MPa. The PVA membranes showed an average Young's modulus of 36.21 ± 8.84 MPa. The observed results are in good agreement with the reported results.^42^ However, the co-spun membranes (PPCS and PPCS-SDF1) showed a comparable elongation at break (70 to 76%), tensile strength (1.8 to 2.1 MPa) and tensile modulus (38 to 42 MPa) to those of the PCL membranes. The reasonably similar tensile values of the PPCS and PPCS-SDF1 membranes suggest that the introduction of SDF1 did not influence the tensile mechanical properties of the membranes.

### 3.3. Hydrophilicity, exudate uptake capacity, and SDF1 release

**Fig. 4:**
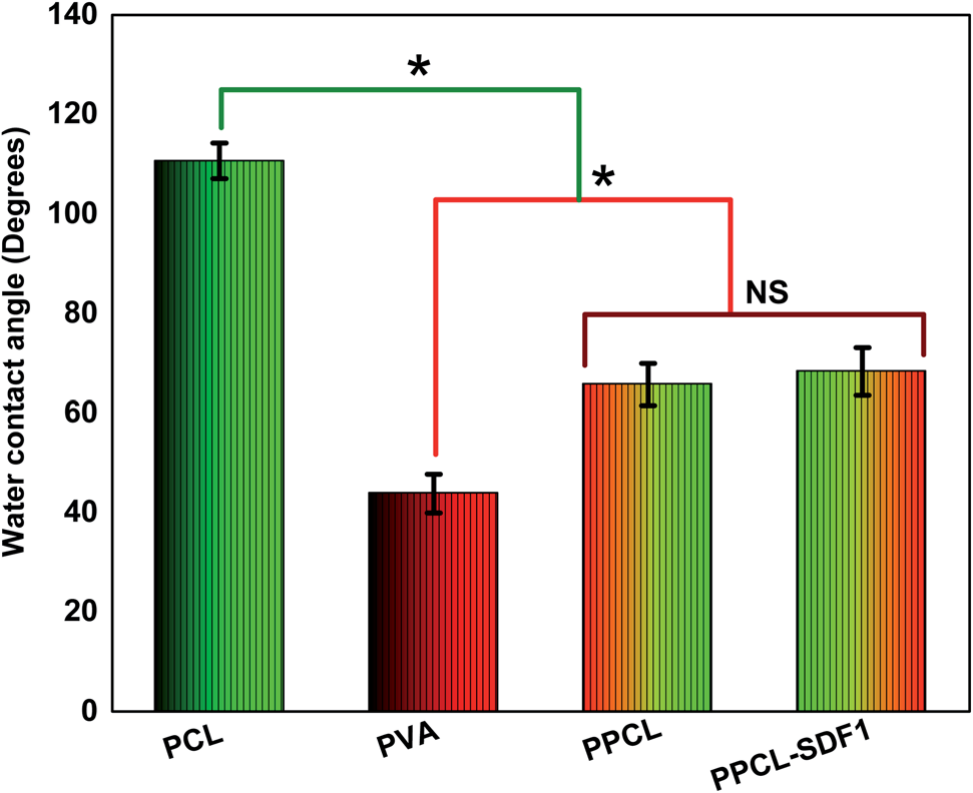
The water contact angles of PCL, PVA, PPCS, and PPCS-SDF1 membranes. (*) indicates *p*-values where a statistically significant difference (*p* ≤ 0.05) is observed. NS indicates no statistically significant difference.

The surface hydrophilicity of the membranes was evaluated by measuring the water contact angle and the findings are summarized in Fig. 4. The single component PCL membranes displayed a high-level water contact angle of 110.7 ± 3.6°, indicating the high surface hydrophobicity.^43^ The single component PVA membranes displayed a contact angle of 43.8 ± 3.9°, indicating a very high hydrophilicity.^44^ Interestingly, the bicomponent PPCS membranes showed a medium water contact angle (65.7 ± 4.2°). This can be ascribed to the presence of hydrophobic PCL fibers and hydrophilic PVA fibers in the co-spun membranes. However, the incorporation of SDF1 did not modify the water contact angle of the co-spun membranes (68.3 ± 4.8°). In addition to the possible desirable performance of a moderately hydrophilic membrane in wound healing, the obtained results also corroborated the successful formation of a hydrophilic/hydrophobic bicomponent membrane.

**Fig. 5:**
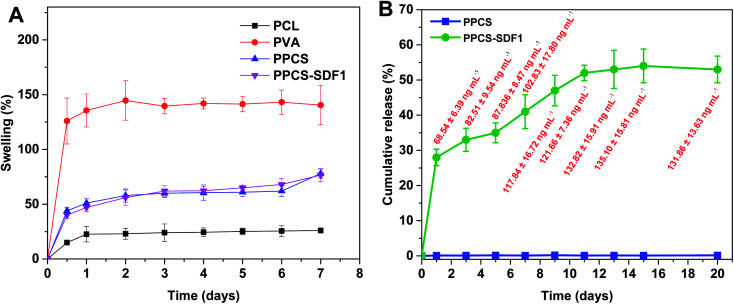
The swelling of membranes (A) and the cumulative release of SDF1 from PPCS-SDF1 membranes (B). The absolute values of released SDF1 are shown as labels (red font) in (B).

The exudate uptake capacity of PCL, PVA, PPCS and PPCS-SDF1 were evaluated from the swelling behaviour in PBS and the results are presented in Fig. 5A. A low water uptake was noted for the neat PCL membranes (25–30%). In contrast, the neat PVA membranes showed a sudden initial swelling. They could reach a peak swelling value within the first day of the study, and then remained almost the same until the end of the study. A gradual increase in the water uptake of PPCS and PPCS-SDF1 was observed, which peaked during 2–3 days of study (74 to 95%). From the results it can be concluded that the PPCS and PPCS-SDF1 membranes showed a high amount of exudate uptake capacity compared to the PCL membranes.

The release profile of SDF1 from the PPCS-SDF1 membranes is shown in Fig. 5B. For tissue regeneration in the wounds, it is very important to keep sustained and prolonged release of SDF1 from the early to late stages of the wound healing process. For 15 days of analysis, sustained and prolonged release of SDF1 was observed. The release study indicated that about 70–130 ng mL^−1^ SDF1 was maintained throughout the experiment. An initial burst release was observed during the first day of the study when compared to subsequent time periods. Nevertheless, the release of SDF1 was maintained throughout the 15 days of the study. The controlled release of SDF1 can be ascribed to the swelling of the PVA, the loosening of its chains, and the consequent release of SDF1.

### 3.4. Cell viability and proliferation

Fibroblasts, keratinocytes, and endothelial cells showed higher cell viability and proliferation upon incubation with PPCS-SDF1.

#### 3.4.1. Live-dead staining

**Fig. 6:**
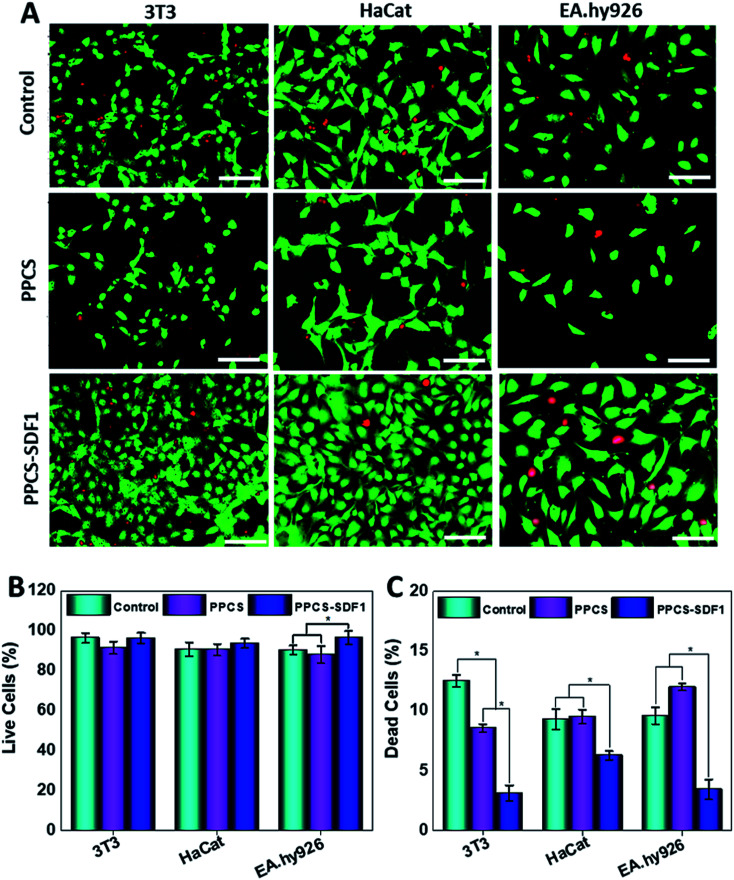
The results of live/dead tests on cells after culturing on PPCS and PPCS-SDF1 membranes. Fluorescent images indicating the viability of mammalian cells that were cultured on PPCS and PPCS-SDF1 membranes (A). Scale bars = 200 μm. The percentages of live cells (B) and dead cells (C) when cultured with the PPCS and PPCS-SDF1 membranes. (*) indicates *p*-values (*p* ≤ 0.05) where a statistically significant difference was detected between compared groups.

Cells in the vicinity of the wound healing membranes should exhibit sufficient viability and proliferation for better healing. Live/dead staining was employed to assess the viability of 3T3 cells, HaCaT keratinocytes and EA.hy296 cells in the presence of the developed membranes. The live and dead cells present on the PPCS and PPCS-SDF1 samples on the 5^th^ day of cell seeding are shown in Fig. 6A. A relatively small number of fibroblast cells were able to grow on the PPCS membranes compared to the PPCS-SDF1 membranes. Several green coloured cells (living cells) were observed on the PPCS-SDF1 membranes when compared to the control and PPCS membranes. A slight reduction in cell proliferation was noted in the PPCS groups compared to the untreated control wells. Similarly, both HaCaT keratinocytes and EA.hy926 endothelial cells showed higher proliferation on the PPCS-SDF1 membranes compared to the PPCS membranes. Although there was a substantial difference in cell number between the different groups (ESI, Table S2†), there was no considerable difference in the relative percentage of viable cells, except for the EA.hy926 endothelial cell treated membranes (Fig. 6B). However, in all the three tested cell lines, the relative percentage of dead cells was significantly less in PPCS-SDF1 groups compared to the other groups (Fig. 6C).

#### 3.4.2. MTT cell viability studies

**Fig. 7:**
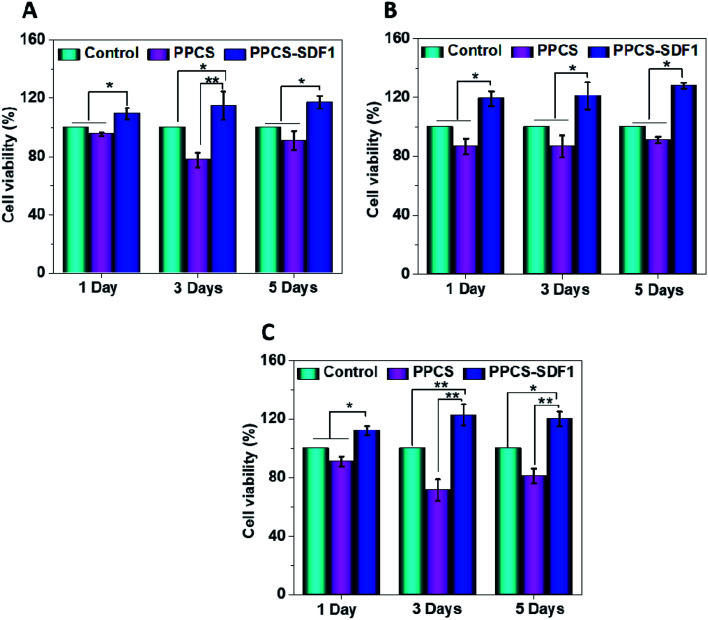
Results of MTT cell viability assays showing the effects of PPCS-SDF1 on the viability of 3T3 (A), HaCaT (B), and EA.hy926 cells (C). (*) indicates *p*-values (*p* ≤ 0.05) where a statistically significant difference was noted between compared groups.

The *in vitro* cell viability of the developed membranes was tested by MTT assay using 3T3, HaCaT and EA.hy926 cells and the obtained results are shown in Fig. 7. PPCS showed a comparable viability to control cells during the 5 days of the study. In the case of all the studied cells, the PPCS-SDF1 membranes showed the highest cell viability compared to the PPCS treated cells and control cells. Compared to the controls, more than 100% relative cell viability (113.5 ± 4.5%) was observed in the case of the PPCS-SDF1 membranes at 24 h and it significantly differed from that of the PPCS-treated cells. Compared to the control cells, the fibroblasts cells cultured with PPCS-SDF1 membranes showed 110.5 ± 4.4%, 116.6 ± 8.4% and 118.8 ± 3.2% viability on day-1, day-3 and day-5, respectively. Compared to the control cells, the keratinocytes cultured with PPCS-SDF1 membranes showed 119.5 ± 7.4%, 121.7 ± 9.8% and 123.8 ± 4.1% viability on day-1, day-3 and day-5, respectively. A relatively comparable trend was noted in the case of the endothelial cells also. These results showed that the percentage viability was independent of the treatment time. The PPCS-SDF1 membranes showed the highest set of viabilities of cells compared with the cells treated with PPCS membranes and the control cells for all three types of cells studied. Overall, the results show that the developed PPCS-SDF1 membranes are cytocompatible and can support the growth of skin-associated mammalian cells *in vitro*.

### 3.5. *In vitro* scratch healing test

**Fig. 8:**
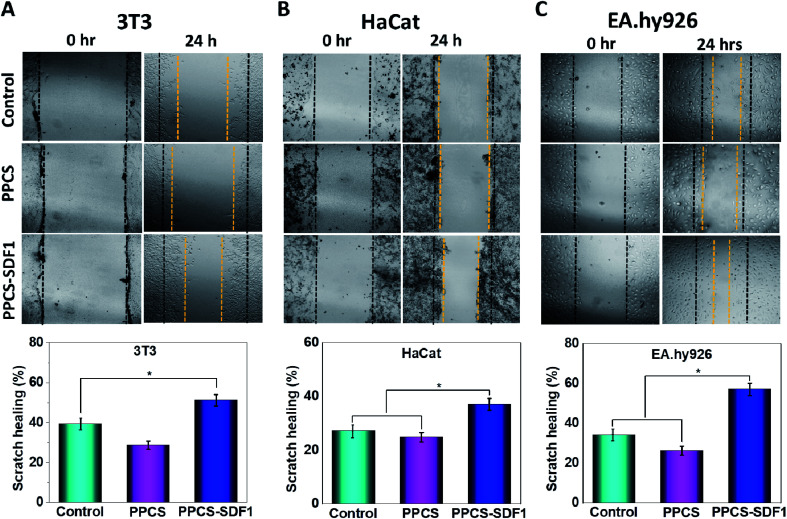
Results demonstrating the effects of PPCS and PPCS-SDF1 membranes on *in vitro* scratch healing using 3T3 fibroblast cells (A), HaCaT keratinocyte cells (B), and EA.hy926 endothelial cells (C). Bar graphs show the scratch contraction (%) after incubation with the developed membranes. (*) indicates *p*-values (*p* ≤ 0.05) where a statistically significant difference was noted between compared groups.

An *in vitro* scratch test was performed to monitor the migration of 3T3 fibroblasts, HaCaT keratinocytes and EA.hy926 endothelial cells, which were cultured with PPCS and PPCS-SDF1 membranes (Fig. 8). A wound contraction of 39.6 ± 8.6% and 25.3 ± 5.4% was noted in the case of the 3T3 cells in the controls and those cultured with PPCS membranes, respectively (Fig. 8A). 3T3 cells treated with PPCS-SDF1 showed a wound contraction of 52.4 ± 9.2%. In contrast, the scratch closure was comparatively slow in the case of HaCaT cells and showed 27.4 ± 3.4% and 25.6 ± 3.8% contraction in the case of the control and PPCS, respectively (Fig. 8B and D). In contrast, the PPCS-SDF1 treated groups showed a wound closure of 43.2 ± 4.2% that was substantially different from that of the PPCS-treated cells and control cells. The ability of the developed membranes to promote the migration of endothelial cells was also investigated. It was observed that the PPCS membrane-treated endothelial cells showed a 28.7 ± 3.4% contraction of the scratch, while the PPCS-SDF1-treated cells showed a scratch closure of 54 ± 6.6% (Fig. 8C, D). The controls showed 33.6 ± 4.8% scratch healing. There was no statistically significant difference in the recovery of the scratches treated with the neat membranes and the controls, whereas the PPCS-SDF1 membranes demonstrated a significant difference in scratch healing.

## 4. Discussion

Electrospun membranes are generally utilized for tissue engineering and wound healing applications with many promising outcomes.^45^ Good elasticity, improved permeability, and morphology that mimics the extra cellular matrix are the important properties that make electrospun membranes suitable for wound coverage applications. One of the disadvantages associated with the electrospinning technique is the loss of activity of less stable biomolecules like growth factors in the organic solvents that are used in standard electrospinning processes. However, this can be avoided with the help of the co-electrospinning technique, where the biomolecules will be protected in a water-soluble polymer matrix which forms one of the components in the bicomponent membranes. In this investigation, we fabricated co-electrospun membranes based on PVA, PCL and SDF1, where SDF1 was loaded in the PVA fibers. SDF1 loaded hydrophilic PVA fibers preserve the bioactivity of SDF1. Hydrophobic and mechanically stable PCL fibers provide stability to the membrane in the wound environment.

From the SEM images, it was observed that the formed membranes have a highly porous architecture (80% porosity was observed by a reported alcohol diffusion method^46^) with submicron fibers. This porous morphology provides sufficient oxygen and water permeability^47^ while providing sufficient bacterial barrier properties that prevent the attack of microbes in the wounds.^48^ Mechanical and elastic behaviour of biomaterials used for wound coverage applications is an important factor to be considered because mechanically nonmatching membranes can create discomfort and pain, and retard healing due to the stress localized in the dressing or in the surrounding native skin during muscular movement. Neat PVA membranes demonstrate relatively low tensile strength, but the presence of PCL fibers provides enough strength to the membranes. The presence of mechanically strong PCL fibers avoided the failure of the weak PVA chains. The addition of SDF1 has no considerable impact on the tensile properties of PPCS. PPCS-SDF1 membranes with sufficient flexibility, elasticity and tensile strength can mechanically fit into the wound bed without producing movement associated issues. Based on the report by Daly and Odland, the tensile properties of the electrospun membranes reported in this study were comparable with those of human skin.^49^ In contrast, another report indicated that the mean ultimate tensile strength of human skin is about 21 MPa,^50^ which was relatively higher than that of the electrospun membranes developed in this study. However, the elongation at break of the membranes developed in this study was comparable with that of human skin (54% for skin).^50^ As the wound healing patches are intended to be used as a temporary protective barrier, it is not very important to have a similar tensile strength to that of human skin. However, it is essential to have enough stress bearing capacity and elasticity to keep the patch in the wound site without failure due to muscular movements.

Successful wound care regimens aim to produce a balanced environment, *i.e.* a moist atmosphere to encourage healing, although not too wet to result in maceration.^51^ It is often not easy to opt for a wound coverage matrix or membrane that is helpful in facilitating healing, eliminates extra exudates and offers protective barrier functions until the wound healing process is completed.^52^ Such matrices should be capable of absorbing and managing the large quantity of exudates generated by chronic wounds.^53^ Moreover, a moderately hydrophilic membrane can provide a better microenvironment for the recruitment and proliferation of native cells.^54^ Thus, we evaluated the hydrophilicity and exudate management capacity of the developed membranes. Contact angle measurements of the samples clearly demonstrated the formation of a moderately hydrophilic membrane upon co-spinning due to the presence of both hydrophobic and hydrophilic fibers. The PCL membrane showed less swelling owing to the presence of hydrophobic functional groups in its chemical composition. The very high absorbing capacity of the PVA membranes was due to the presence of amorphous regions in the PVA, because it is less ordered and more available for water.^55^ Supporting evidence from other investigations on PVA–clay nanocomposites illustrates a similar trend as obtained in this investigation.^56^ However, the application of PVA membrane-based wound coverage matrices with large water holding capacity in the wound can result in maceration.^57^ PPCS has the optimum ability to hold an excess amount of exudate, which allows it to act as a suitable candidate for even highly exudative wounds.^58^ Moreover, the presence of hydrophobic surfaces shows reduced cell attachment, whereas surfaces with reasonable hydrophilicity facilitate cell adhesion and proliferation.^59^ Thus, the PPCS-SDF1 membranes can promote wound healing by ensuring a suitable wound microenvironment. To ensure both speed and quality of wound healing, loaded SDF1 should be released in a sustained manner until the wound is completely healed. The quick swelling of the PVA fibers resulted in an initial burst release of SDF1 as corroborated from earlier studies.^60^ However, the slow release of SDF1 form the PPCS-SDF1 membrane during the subsequent period ensures appropriate cellular responses and results in quick wound healing. The results obtained in this study indicate that about 70–130 ng mL^−1^ SDF1 released from the patches was sufficient to support cell proliferation and cell migration, which may help in wound healing. Earlier studies also showed that nanogram to sub-microgram per mL quantities of SDF1 were sufficient to promote cell proliferation and wound healing.^15,19,61^

Besides several biological effects, SDF1 participates in a number of key mechanisms that will trigger and protect mammalian cells from undergoing apoptosis or necrosis.^62^ Earlier studies demonstrated that SDF1 treatment can support cell survival and differentiation as a result of the increased expression of relevant proteins such as Runx-2 mRNA, and ALP and Runx-2.^63^ This protective effect of SDF1 was clearly observed in the enhancement of the viability of fibroblast cells seeded on SDF1-loaded membranes. A very similar behaviour was noted in the case of HaCaT and endothelial cells cultured on SDF1-incorporated membranes. Enhanced proliferation of the cells treated with the PPCS-SDF1 membranes could be associated with the gradual release of SDF1 from the PVA fibers. The effect of the developed membranes on the migration of wound healing-associated cells was also investigated. We observed a rapid contraction of *in vitro* wounds created on cell culture plates which were treated with PPCS-SDF1 membranes. This was due to the higher migration of the cells from the scratch boundaries to the cell free area as a result of the effect of SDF1 released from the fibers.^64^ Cell migration from the wound margins is an important factor for the re-epithelialization of wounds, and hence the SDF1-loaded membranes can be considered as a suitable material for the development of diabetic wound healing membranes.^18^

The results gathered from the present investigation demonstrate that SDF1-loaded membranes could improve the proliferation and viability of mammalian cells such as fibroblast cells, keratinocyte cells and endothelial cells, which are very relevant during the process of wound healing. The co-spun membranes loaded with an appropriate amount of SDF1 promoted wound contraction in an *in vitro* model due to the sustained release of SDF1. Future studies should aim at the *in vivo* evaluation of the effect of SDF1-loaded co-spun membranes on cell proliferation, vascularization and wound closure in small animal models.

## 5. Conclusions

In this study, co-electrospun SDF1-loaded polymeric membranes were fabricated and systematically characterized. The morphological features of the developed membranes were examined *via* SEM analysis. Tensile testing, swelling studies, and cumulative release studies were also performed to understand the mechanical strength, exudate uptake capacity, and SDF1 release from the co-spun membranes, respectively. Detailed *in vitro* cell culture studies were performed to evaluate the proliferation and viability of skin-wound-healing-associated cells, such as fibroblasts, keratinocytes, and endothelial cells. The *in vitro* wound contraction potential of the membranes was also examined. The results of SEM analysis indicated that the fabricated membranes comprised both PCL and PVA fibers. The fabricated membranes possessed suitable flexibility, tensile strength, and modulus values for use in the development of wound-healing products. The SDF1-laden membranes offered higher cell viability and facilitated the excellent proliferation of mammalian cells, such as 3T3 fibroblasts, HaCaT keratinocytes, and EA.hy926 endothelial cells. The SDF1-loaded membranes also resulted in higher scratch wound healing *in vitro*. The results obtained in the current investigation suggest that SDF1-loaded membranes could be applied for promoting wound healing, especially in diabetic patients. However, future studies should focus on detailed *in vivo* experiments to invariably demonstrate the biocompatibility and wound-healing potential of the membranes developed in this research.

## Conflicts of interest

The authors declare that they have no known competing financial interests or personal relationships that could have appeared to influence the work reported in this paper.

## Supplementary Material

RA-011-D0RA04997B-s001
