# Supplementary material for: Stromal cell-derived factor loaded co-electrospun hydrophilic/hydrophobic bicomponent membranes for wound protection and healing
Source: RSC Adv. 2020 Dec 24;11(1):572–83. doi: 10.1039/d0ra04997b (PMC8691117; doi:10.1039/d0ra04997b)
Supplement: RA-011-D0RA04997B-s001 [file RA-011-D0RA04997B-s001.pdf]

Electronic Supplementary Material (ESI) for RSC Advances.
This journal is © The Royal Society of Chemistry 2020

Stromal cell-derived factor loaded co-electrospun hydrophilic/hydrophobic bicomponent membranes for wound protection and healing

Robin Augustine[1,2], Syed Raza ur Rehman[1,2], Joshy K.S[1,2], Anwarul Hasan[1,2]*

[1]Department of Mechanical and Industrial Engineering, College of Engineering, Qatar University–2713, Doha, Qatar

[2]Biomedical Research Center, Qatar University– 2713, Doha, Qatar

Corresponding author Email: ahasan@qu.edu.qa

Optimization of required SDF concentration in PPCS

We have fabricated electrospun PVA membranes loaded with 0.05% w/w, 0.075% w/w, 0.1% w/w, 0.125% w/w and 0.15% w/w SDF with respect the weight of PVA. Experimental procedures used for the development of SDF loaded PVA membranes and crosslinking were similar to that of PPCS-SDF the main manuscript except that PCL was not co-spun here and we used only 3 mL solution for electrospinning to minimize the use of expensive SDF.

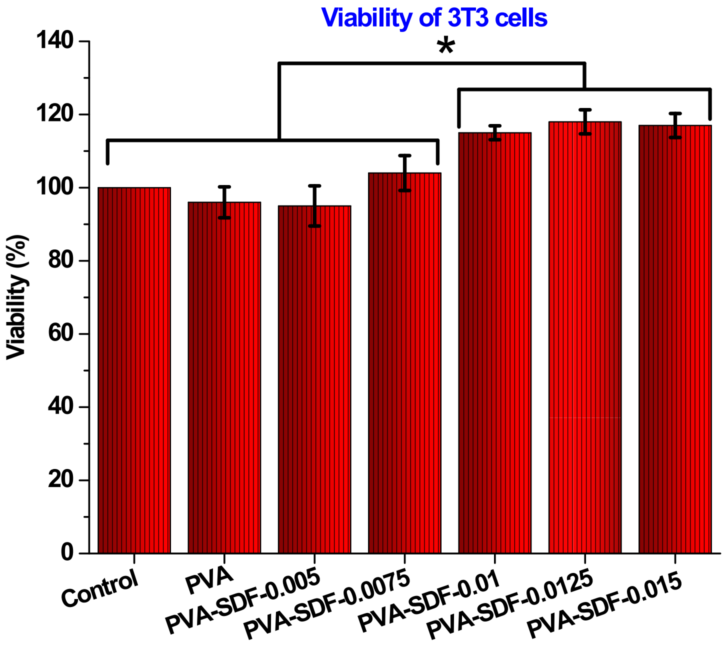

Figure S1. MTT assay result indicating the viability of 3T3 fibroblasts which were grown in the presence of patches containing various amounts of SDF for 48 h. (*) indicates the P-values where a statistically significant difference ($p \leq 0.05$) is observed.

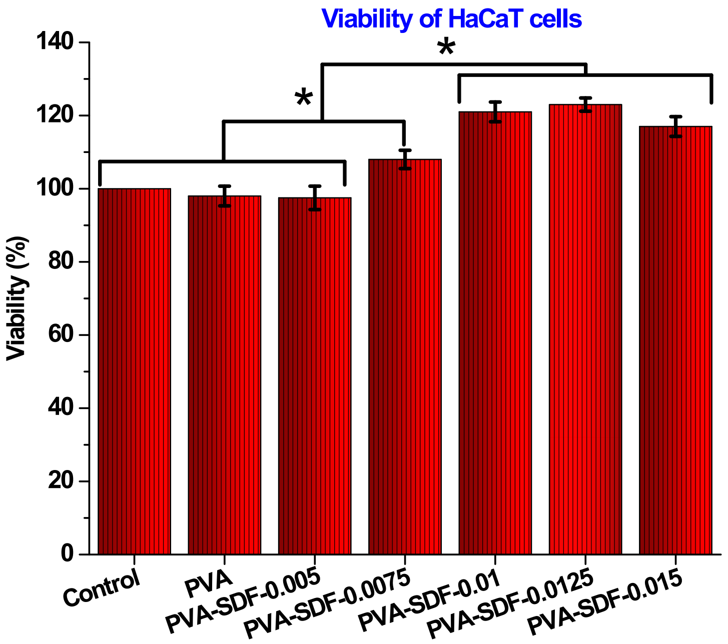

Figure S2. MTT assay result indicating the viability of HaCaT Keratinocytes which were grown in the presence of PVA membranes containing various amounts of SDF for 48 h. (*) indicates the P-values where a statistically significant difference ($p \leq 0.05$) is observed.

Method of quantification of proportion of PVA and PCL in the PPCS

Percentage of PVA and PCL fibers in the PPCL samples were determined by specifically removing PCL from the samples. 500 mg of PPCS samples were placed in acetone (10 mL) in a glass bottle with magnetic stirring (200 rpm) for 24 h. Then, the samples were taken out from acetone, rinsed 3 times in acetone to remove adsorbed PCL and air dried for 24 h. Dried samples were reweighed to find out the percentage of PVA (Eq. 1) and PCL (Eq. 2) in the samples.

$$Percentage\ of\ PVA = \left(\frac{Weight\ of\ sample\ after\ removal\ of\ PCL}{Weight\ of\ sample\ before\ removal\ of\ PCL}\right) \times 100 \quad (1)$$

$$Percentage\ of\ PCL = 100 - Percentage\ of\ PVA \quad (2)$$

Table S1: Results of quantification of proportion of PVA and PCL in the PPCS

Sample	PVA	PCL
PPCS sample 1	44.86	55.14
PPCS sample 2	40.58	59.42
PPCS sample 3	46.75	54.25
Average	44.06 ± 3.16	56.27 ± 2.76

Quantification of cells from fluorescence images

Number of live and dead cells were quantified from fluorescent images. Images taken using 10X objective was used for the quantification. Minimum three different focus areas were used for counting the cells.

Table S2: Quantification of cells from fluorescence images

Sample	3T3			HaCat			EA.hy926		
	Live	Dead	Total	Live	Dead	Total	Dead	Dead	Total
Control	108 ± 5.3	14 ± 2.1	122 ± 8.7	78 ± 7.4	8 ± 2.5	86 ± 2.8	47 ± 1.9	5 ± 1.7	52 ± 3.2
PPCS	64 ± 4.4	6 ± 3.6	70 ± 9.2	57 ± 7.3	6 ± 3.8	63 ± 4.4	22 ± 2.2	3 ± 3.2	25 ± 6.8
PPCS-SDF1	184 ± 8.2	17 ± 4.3	201 ± 12.5	134 ± 11.4	9 ± 2.3	143 ± 13.7	56 ± 4.5	2 ± 2.3	58 ± .8.8
